# Developing the latest framework to measure and incentivise pharmaceutical industry contributions to health research and development

**DOI:** 10.1186/s12961-018-0332-y

**Published:** 2018-08-02

**Authors:** Clarke B. Cole, Stine Trolle, Danny J. Edwards

**Affiliations:** Access to Medicine Foundation, Naritaweg 227A, 1043 CB Amsterdam, The Netherlands

**Keywords:** Research and development, Medicine, Access, Methodology, LMIC, Incentive, Health priorities, Research, Drug industry, Global Health

## Abstract

Major pharmaceutical companies contribute important expertise to health research and development (R&D), particularly in their ability to develop and bring pharmaceuticals to market. The Access to Medicine Index evaluates how 20 of the world’s largest pharmaceutical companies are directing R&D efforts towards the needs of people living in low- and middle-income countries. In dissemination of its findings, the Index stimulates pharmaceutical companies to expand R&D activities in this direction. The Index methodology is reviewed every 2 years, most recently for the 2018 Index, to ensure their R&D activity is benchmarked against current health R&D priorities as defined by the global health community. The review is based on consensus-building processes involving global health stakeholders. In the latest review, two main changes to the methodology were made that will further deepen the Index’s analysis of (1) how far companies’ R&D activity aligns with global health priorities; and (2) whether companies make plans to ensure resulting innovations reach populations in need globally. These changes will be applied in the 2018 Access to Medicine Index. Importantly, the methodology review process highlighted the need for further prioritisation from the global health community, in particular to emphasise to innovators which product innovations are needed most critically to address the burden of non-communicable diseases in low- and middle-income countries. Should such prioritisations be developed, the Index can play an important role in tracking and stimulating company contributions towards them.

**Electronic supplementary material:**

The online version of this article (10.1186/s12961-018-0332-y) contains supplementary material, which is available to authorized users.

## Background

Private sector contributions make up 60% of health research and development (R&D) investments in high-income countries [1]. Major pharmaceutical companies, largely based in these countries, contribute significant resources to health R&D, including the capacity to develop and bring pharmaceuticals to market. Due to lower commercial market potential, these companies can face lower incentives to engage in R&D that specifically addresses the needs of populations in low- and middle-income countries (LMICs).

The Access to Medicine Foundation is an independent, non-profit organisation that works to stimulate the pharmaceutical industry to make greater efforts to address the needs of populations in LMICs. Integral to the Foundation’s work in this regard is its Access to Medicine Index (ATMI), which measures, compares and ranks the top 20 of the world’s largest (by market capitalisation) R&D-based pharmaceutical companies on their efforts to improve access to medicine in LMICs.

The methodology for the ATMI involves a tool for identifying and expanding good practice across the pharmaceutical industry. It uses a weighted set of metrics to assess company action to improve access to medicine for high-burden and priority diseases in LMICs (in the 2018 ATMI, 77 diseases and 106 countries were included). Company activities are measured across topical areas, including access governance, pricing, intellectual property management and R&D.

In order to keep pace with new priorities and evolving expectations of companies in access to medicine, the Access to Medicine Foundation reviews and updates the ATMI methodology every 2 years using a multi-stakeholder consensus building process. This includes a review and ratification of each updated methodology by an independent Expert Review Committee, comprised of representatives from WHO, governments, non-governmental organisations, patient organisations, the pharmaceutical industry, academia and investors. The methodology for the 2018 ATMI, published in October 2017, represents current expectations for pharmaceutical company behaviour [2]. The next iteration of the ATMI, to be published in 2018, will evaluate companies on the extent to which they meet these expectations, aiming to stimulate a race to do well and identifying new insights into the best approaches for driving change.

Overall, 20% of the ATMI analysis focuses on companies’ R&D activities. The R&D analysis measures a range of areas relevant to access to medicine grouped under the following main subthemes: product development (which includes metrics such as the level of R&D investment in diseases within scope of the ATMI and the movement of R&D projects along the pipeline), planning for access during R&D and ethical clinical trial conduct. Following publication of the 2016 ATMI [3], the metrics used to measure companies’ R&D activities were reviewed by the Access to Medicine Foundation, with the support of external technical experts in pharmaceutical R&D. Companies also provided feedback on the relevance of the 2016 metrics to company activities, and their ability to drive change in this regard. Additionally, a range of global health experts were consulted on specific issues and expectations of companies to improve access via R&D. As a result of this process, decisions were taken to retain existing measures and to implement select changes to how the ATMI will measure companies on their activities to improve access to medicine.

Key changes to the methodology that specifically relate to how companies are expected to support access through R&D are (1) whether R&D projects address a comprehensive set of global health priorities, and (2) whether companies put plans in place to ensure that successful innovations are rapidly made accessible in LMICs following first global marketing approval.

These changes aim to stimulate pharmaceutical companies to more closely align their R&D activities with the needs of LMIC populations, from defining their R&D strategies and targets to making newly approved products accessible to populations in need globally. This paper explores the context and rationales for these changes in order to highlight the value of the ATMI methodology in tracking industry contributions to priorities for global health R&D.

### Setting R&D priorities to ensure access in LMICs

Global health R&D priorities represent the R&D most urgently needed to address risks to the health of the global population. Often, they specifically focus on the need for pharmaceutical R&D. R&D priority lists defined by global health stakeholders, such as WHO and Policy Cures Research, have prioritised pharmaceutical R&D that addresses a range of global health challenges, from those that disproportionately affect populations in LMICs (e.g. HIV/AIDS, malaria, tuberculosis and neglected tropical diseases) to those that threaten health security at a global scale (e.g. emerging infectious diseases and antibiotic-resistant bacteria). Such prioritisations aim to spur R&D in instances where no products are available to address these risks, or where existing products are suboptimal or specifically unsuitable for use in certain settings such as where resources are limited. R&D priorities are critical inputs to direct stakeholder engagement, including industry engagement, in global health R&D, in particular where commercial market incentives are low.

**Fig. 1:**
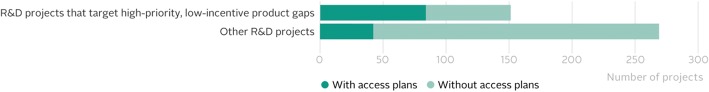
The 2016 Access to Medicine Index found that the 20 companies measured had 420 projects in development targeting the needs of people in low- and middle-income countries (LMICs). Of these, 36% of projects (151/420) targeted a research and development (R&D) priority and 30% (126/420) had access plans in place, two-thirds (67% or 85/126) of which also targeted an R&D priority. To drive the industry further to ensure populations in LMICs gain rapid access to the most-needed products, the 2018 Index will deepen analysis on both criteria [3]

R&D prioritisation not only helps to spur R&D according to the needs of populations in LMICs. There is also an apparent relationship between targeting R&D priorities and putting plans in place as early as possible to support rapid access to newly approved products by these populations. In 2016, the ATMI found that R&D projects targeting R&D priorities were more likely to have access plans in place than those not targeting R&D priorities (Fig. 1). In 2016, companies measured by the ATMI had 420 R&D projects, 36% of which (151/420) targeted a R&D priority set by Policy Cures Research G-FINDER tool (e.g. a product gap where there is urgent need but low commercial incentive to engage in R&D) [4, 5]. Further, 30% (126/420) of companies’ R&D projects had access plans in place, 67% (85/126) of which also targeted an R&D priority.

The relationship between R&D prioritisation and access planning may be explained by several factors, one of which is the influence of public and philanthropic research partners with whom companies tend to collaborate when developing products for R&D priorities (usually through product development partnerships). Indeed, the 2016 ATMI found that products developed in partnership included access provisions earlier and more often than those developed in-house. A further factor is the simple reality that, if commercial market potential is low, as is often the case where R&D priorities exist, the market alone cannot ensure populations in need will be able to access newly approved products. This may be for a variety of reasons, ranging from low incentives for companies to register products in jurisdictions with small markets, to products being available but priced at levels that are unaffordable to those in need. Limited health system infrastructure can compound this issue. As such, planning for access is an important part of product development in these cases.

To increase the capacity of the ATMI to incentivise companies to conduct R&D that aligns closely with the most pressing global health needs, and which supports rapid access to the resulting products for those in need, the 2018 ATMI will deepen analysis on how companies address both these factors within their R&D activities.

## Responding to priorities for global health R&D

**Fig. 2:**
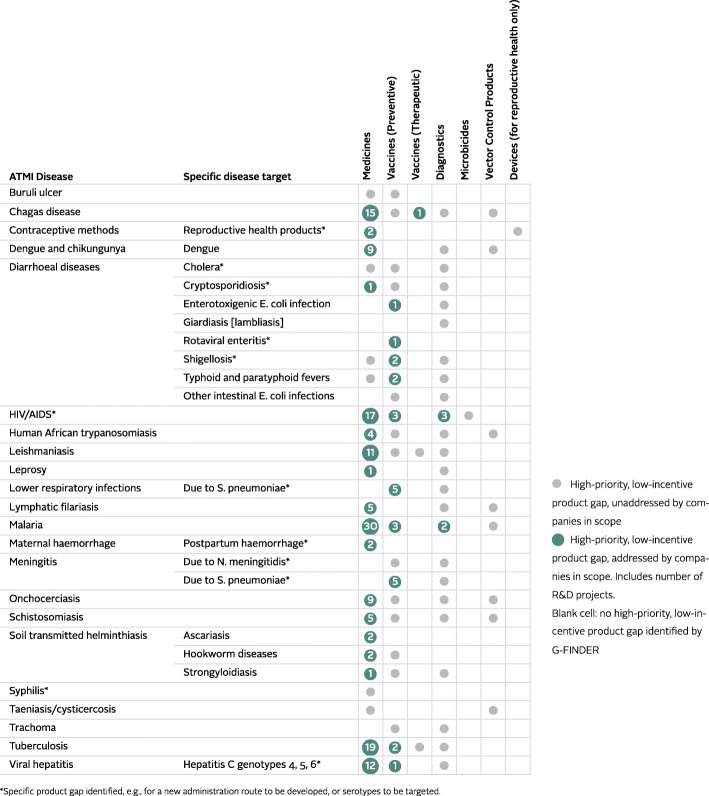
The 2016 Index found companies were conducting research and development (R&D) for 31 out of 84 (37%) high-priority R&D gaps with low commercial incentives. Projects that target multiple diseases, or are being co-developed by more than one company, are counted more than once

R&D-based pharmaceutical companies play an important role in addressing R&D priorities. In 2016, the ATMI found that companies measured were addressing 31 out of 84 high priority R&D gaps for neglected diseases and reproductive health in areas where low commercial incentives exist, as defined by Policy Cures Research’s G-FINDER tool [4, 5]. The priorities that received the most attention in 2016 were for malaria (35 R&D projects), followed by HIV/AIDS (23 R&D projects) and tuberculosis (21 R&D projects) (Fig. 2). This is consistent with G-FINDER data on financial investments in R&D for neglected diseases; as in previous years, in 2016, these three diseases collectively received over two-thirds (USD 2247 million or 70%) of all global funding for neglected disease R&D [6].

However, other R&D priorities set by G-FINDER, such as contraceptives and products for maternal haemorrhage and syphilis, were found to be largely neglected by the companies measured. This is concerning, as maternal health conditions place an immense burden on LMICs in the research scope of the ATMI^1^; indeed, 99% (82,447/83,126) of mortality due to maternal haemorrhage occurs in these countries [7]. The same LMICs carry 99% (7,946,266.36/8,065,237.63) of the global DALY burden for syphilis [8].

The 2016 ATMI found that six out of 20 companies evaluated accounted for the majority of R&D targeting the priorities set out by G-FINDER. However, all R&D-based pharmaceutical companies have a responsibility to contribute to R&D that addresses the specific needs of populations in LMICs.

## Driving the industry further in priority R&D

To help stimulate the industry to increase engagement in R&D that aligns with the needs of populations in LMICs, the 2018 ATMI methodology has expanded to include a broader set of global health R&D priorities. The list of prioritisations, identified through literature review and expert validation, retains Policy Cures Research’s G-FINDER neglected diseases and reproductive health areas (which were also used in the 2016 ATMI) and now includes (1) the WHO priority pathogens list for R&D of new antibiotics, (2) priorities set out by the WHO Initiative for Vaccine Research and (3) the WHO R&D Blueprint. The prioritisation lists define specific R&D needs and product gaps for populations in LMICs, as well as gaps linked to potential global health threats. As a result, the 2018 ATMI will now evaluate companies on the extent to which they are conducting R&D that addresses R&D priorities for 45 diseases and pathogens, up from 22 in 2016 (refer to Additional file 1 for a full list of these priorities) [2]. By expanding to include additional lists, the ATMI will now measure companies on their efforts to develop vaccines that address a more comprehensive range of needs specific to people living in LMICs, as well as their contributions to combatting antimicrobial resistance and the risks of emerging infectious diseases through R&D.

In some cases, global health stakeholders have identified the same R&D priorities in different priority lists, stressing their importance on multiple factors relevant to global health. For example, Zika has been identified on two of the R&D priority lists. The WHO Initiative for Vaccine Research has identified a need for a preventive vaccine to protect women of reproductive age from Zika infection in an outbreak context [9]. This prioritisation is based on the relevance of the disease to LMICs and the potential for outbreaks to be controlled via immunisation [9]. R&D for Zika has also been prioritised by the WHO R&D Blueprint due to the epidemic potential of the disease along with the lack of sufficient countermeasures against it [10].

By reviewing the literature and consulting experts, it also became evident that global health priorities for R&D have not been defined for non-communicable diseases (NCDs), despite the growing burden of NCDs globally and that the majority of global NCD burden is borne by LMICs. For example, 86% of global premature mortality due to NCDs occurs in LMICs [11]. Given this, a country-level review that identifies the range of needs for new and adapted products that specifically address barriers to access is required. Prioritisation of R&D for NCDs will be critical to support coordinated efforts by all stakeholders, including the private sector, to address such access needs.

## Planning to ensure access to successful innovations

Planning for access as early as possible during R&D allows companies to work towards achieving product characteristics that directly respond to needs in low-resource settings. It also facilitates more rapid deployment of successful innovations to LMICs. For example, by setting registration targets early, a company can better ensure clinical testing is conducted in the jurisdictions required to enable efficient registration in those markets. In another example, by making commitments during R&D to price a product affordably in LMICs, a company can work towards a cost of goods low enough to set a price that is sustainable for both itself and the health systems that will procure it. The combination of access provisions required to ensure access in LMICs will vary depending on the characteristics of the product candidate in question, as well as the nature of the health system and commercial markets in which it will be delivered.

**Fig. 3:**
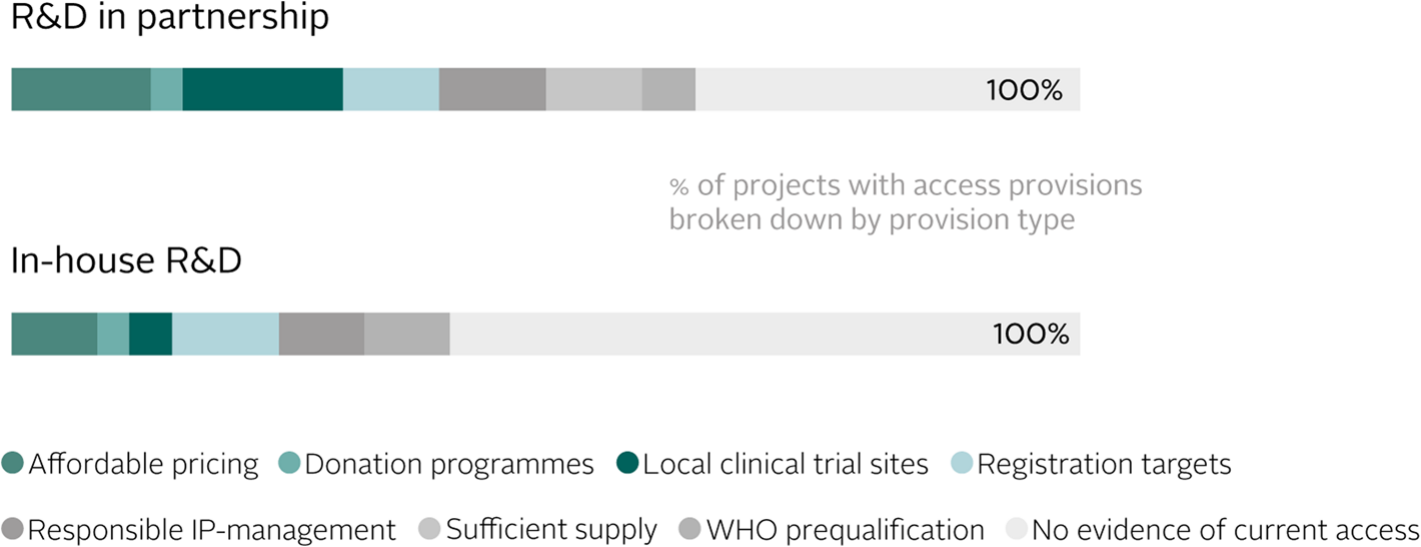
The 2016 Index found that late-stage research and development projects conducted in partnership included at least one access provision more often than in-house projects (63% versus 41% of projects, respectively). Companies reported seven types of access provisions that aim to address a range of barriers to access in low- and middle-income countries [3]

The 2016 ATMI found that R&D projects conducted in-house included access provisions less often than those conducted in partnership, signalling that collaborative models influence the incorporation of access provisions in R&D projects (Fig. 3). The ATMI has also found that companies tend to conduct R&D collaboratively far more often for communicable diseases and neglected tropical diseases than for NCDs. It follows from this that some groups of products are largely left without access planning. For example, almost all R&D projects for NCDs in 2016 were conducted in-house (94%), and only 11% included at least one access provision. This is concerning as populations in LMICs, which face a high and growing burden of NCDs, have a pressing need for rapid access to appropriate products for NCDs.

The lack of collaboration and access planning in R&D for NCDs can be explained by the substantial commercial market potential that drives such R&D. Since the burden of NCDs is substantial in countries at all income levels, companies tend to focus on bringing new NCD products to markets with the greatest abilities to pay for them. This means that, while market incentives may drive R&D for NCDs, there is no guarantee that these products will have the characteristics required to meet the needs of LMIC populations. Further, planning for access in LMICs may be a lower priority than reaching high-income country markets. R&D collaborations between companies and public or non-governmental organisations are rare for NCDs because R&D incentives that use a shared-risk model, such as product development partnerships, have not been considered necessary to ensure companies conduct R&D for NCDs. In turn, the positive influence of such models on access planning is so far lacking in the NCD space.

To incentivise companies to close the gap in access planning, we gathered perspectives from eight prominent global health organisations with expertise in R&D on the importance of planning for access during R&D. The resulting 2018 ATMI methodology now incentivises companies to have access provisions in place for R&D projects regardless of whether they are conducted in-house or in partnership. Emphasis will be placed on projects in late stages of development (phase II clinical trials or later) by conducting a deeper analysis into the nature of access plans for product candidates that are closest to approval. However, companies are encouraged to develop access plans as early in development as possible – the earlier a company considers which products are needed most and by whom, the better it will be able to tailor a product candidate’s characteristics to meet the specific needs of the target population. Further, planning for access early makes it more likely that these plans will ensure rapid access to the product by the target populations. The results of this analysis will shed light on the extent to which companies plan to make their R&D candidates – across all disease areas, and regardless of whether they are working in partnerships – accessible in LMICs upon approval.

## Conclusions

The biennial review of the ATMI methodology, marked most recently by the 2018 ATMI methodology, provides an opportunity to ensure the pharmaceutical industry is measured against current health R&D priorities. The process ensures companies can be tracked, using the ATMI, on their contributions to health R&D. This includes highlighting the areas in which companies are active, and stimulating engagement where their activities are limited or absent.

The methodology draws on the most current consensus among global health stakeholders on how the R&D-based pharmaceutical industry is expected to contribute to global health R&D.

This is reflected in particular by the two changes to the methodology described here. These changes are designed to drive change in how the industry addresses access through R&D. First, the list of R&D priorities against which companies will be measured has been expanded, incentivising companies to align their R&D activities more closely with global health needs. Second, companies will no longer be evaluated on whether they plan for access only in collaborative R&D projects; access planning will now be evaluated for all R&D projects. As companies tend to conduct R&D collaboratively in areas where market incentives are low, this is an important means to incentivise access planning for all needed products, even those for which the market drives R&D.

The methodology review process clarified the need for further prioritisation from the global health community. This includes R&D prioritisation for disease areas like NCDs, where commercial market incentives exist but do not guarantee the most highly needed products, nor that newly approved products will have access plans in place. Such prioritisation should emphasise to innovators which product innovations are most critical to address the burden of NCDs in LMICs, meanwhile stressing the importance of early planning for access. Should such prioritisations be developed, the ATMI can play an important role in tracking and stimulating company contributions towards them.

## Additional file

Additional file 1: Priority diseases and pathogens for research and development analysis of the 2018 Access to Medicine Index. (PDF 88 kb)

## Abbreviations

ATMI: Access to Medicine Index
LMICs: Low- and middle-income countries
NCDs: Non-communicable diseases
R&D: Research and development

## Footnotes

1 The Access to Medicine Index measures pharmaceutical companies’ actions in countries where greater access to medicine is most needed. These countries are identified using three criteria, namely (1) countries’ levels of income (gross national income per capita); (2) their levels of development; (3) and the scope and scale of inequality in each country.

## References

[1] Rottingen JA, Regmi S, Eide M, Young AJ, Viergever RF, Ardal C, Guzman J, Edwards D, Matlin SA, Terry RF (2013). Mapping of available health research and development data: what’s there, what’s missing, and what role is there for a global observatory?. Lancet.

[2] Access to Medicine Foundation (Research team includes Cole CB, Edwards DJ, Trolle S). The 2018 Access to Medicine Index Methodology. Access to Medicine Foundation. 2017. https://accesstomedicineindex.org/media/atmi/2017-Methodology-2018-Access-to-Medicine-Index.pdf. Accessed 25 Apr 2018.

[3] Access to Medicine Foundation (Research team includes Cole CB, Edwards DJ). The Access to Medicine Index 2016. Access to Medicine Foundation. 2016. http://accesstomedicineindex.org/media/atmi/Access-to-Medicine-Index-2016.pdf. Accessed 25 Apr 2018.

[4] Policy Cures. “Neglected Disease R&D Matrix: G-FINDER Diseases, Products and Technologies.” 2015. Accessed 25 Apr 2018 https://gfinder.policycuresresearch.org/staticContent/pdf/Y10-2017_R&D_Matrix_Neglected_Disease_PCR.pdf

[5] Policy Cures. “Reproductive Health R&D Matrix: Reproductive Health Areas, Products and Technologies.” 2014. Accessed 25 Apr 2018 https://gfinder.policycuresresearch.org/staticContent/pdf/G-FINDER-reproductive-product-matrix.pdf

[6] Chapman N et al. Neglected Disease Research and Development: Reflecting on a Decade of Global Investment. Policy Cures Res. 2017. http://policycuresresearch.org/downloads/Y10_G-FINDER_full_report.pdf. Accessed 25 Apr 2018.

[7] Institute for Health Metrics and Evaluation (IHME). GBD Results Tool. 2015. http://ghdx.healthdata.org/gbd-results-tool. Accessed 25 Apr 2018.

[8] World Health Organization. Estimates for 2000–2015. In: Health Statistics and Information Systems. World Health Organization. 2018. http://www.who.int/healthinfo/global_burden_disease/estimates/en/index2.html. Accessed 25 Apr 2018.

[9] World Health Organization. Zika virus vaccine product development. In: Immunization, Vaccines and Biologicals. World Health Organization. 2018. http://www.who.int/immunization/research/development/zika/en/. Accessed 25 Apr 2018.

[10] World Health Organization. List of blueprint priority diseases. In: R&D Blueprint. World Health Organization. 2018. http://www.who.int/blueprint/priority-diseases/en/. Accessed 25 Apr 2018.

[11] World Health Organization. Global Action Plan for the Prevention and Control of NCDs 2013–2020. 2013. http://apps.who.int/iris/bitstream/handle/10665/94384/9789241506236_eng.pdf?sequence=1. Accessed 25 Apr 2018.

