# Supplementary material for: Developing the latest framework to measure and incentivise pharmaceutical industry contributions to health research and development
Source: Health Res Policy Syst. 2018 Aug 2;16:73. doi: 10.1186/s12961-018-0332-y (PMC6090890; doi:10.1186/s12961-018-0332-y)
Supplement: Supplementary file 1 — Priority diseases and pathogens for research and development analysis of the 2018 Access to Medicine Index. (PDF 88 kb) [file 12961_2018_332_MOESM1_ESM.pdf]

ADDITIONAL FILE 1. PRIORITY DISEASES AND PATHOGENS FOR R&D ANALYSIS

Priority diseases and pathogens analysed in the Research & Development Technical Area

The 2018 Access to Medicine Index has placed further emphasis on R&D for projects that address specific priority product gaps. The table below provides an overview of the criteria and priority lists used to identify diseases with R&D priority gaps. The diseases in scope for R&D include 45 (out of 77) diseases with an identified priority product gap.

Some diseases are included in more than one priority list. Pathogens have been brought into the disease scope for the 2018 Index for the first time. These have been identified by WHO on its pathogen priority list: as priority R&D targets for new and effective antibiotics active against the pathogens themselves and the diseases they cause. This WHO pathogen priority list does not define specific products needed.

ATMI Disease	Specific disease target	Medicines	Vaccines (Preventive)	Vaccines (Therapeutic)	Diagnostics	Microbicides	Vector Control Products	Devices (for reproductive health only)	Policy Cures Research G-FINDER neglected diseases	Policy Cures Research G-FINDER reproductive health areas	WHO R&D Blueprint	WHO Initiative for Vaccine Research gaps	WHO Pathogen Priority List	Also analysed in other TAs
Arenaviral haemorrhagic fevers	Lassa Fever	●	●	●	●	●					◉			
Buruli ulcer		●	●		●				◉					◉
Chagas disease		●	●	●	●		●		◉					◉
Contraceptive methods	Reproductive health products1	●						●		◉				◉
Crimean Congo Haemorrhagic Fever (CCHF)		●	●	●	●	●	●				◉			
Dengue		●	●		●		●		◉			◉		◉
Diarrhoeal diseases	Cholera	◉	●		●				◉					◉
	Cryptosporidiosis	◉	●		●				◉					◉
	Enterotoxigenic E.coli (ETEC)		●		●				◉					◉
	Giardiasis [lambliasis]				●				◉					◉
	Shigellosis	◉	●		●				◉					◉
	Rotaviral enteritis		◉						◉					◉
	Enteroaggregative E.coli (EAggEC)		●		●				◉					◉
	Typhoid and paratyphoid fever (S. typhi, S. paratyphi A)	●	●		●				◉					◉
	Non-typhoidal S. enterica (NTS)	●	●		●				◉					
Filoviral diseases	Ebola	●	●	●	●	●	●				◉			
	Marburg	●	●	●	●	●	●				◉			
Henipaviral diseases	Nipah	●	●	●	●	●					◉			
HIV/AIDS		◉	●	◉	●	●	●		◉			◉		◉
Human African trypanosomiasis		●	●		●		●		◉					◉
Leishmaniasis		●	●	●	●				◉					◉
Leprosy		●			●				◉					◉
Leptospirosis					◉				◉					
Lower respiratory infections	*S. pneumonia*		◉		●				◉					◉
	Severe Acute Respiratory Syndrome (SARS)	●	●	●	●	●	●				◉			◉
	Influenza		●									◉		◉
	Respiratory syncytial virus (RSV)		●									◉		◉
Lymphatic filariasis		●			●		●		◉					◉
Malaria		●	●		●		●		◉			◉		◉
Maternal haemorrhage	Postpartum haemorrhage	◉								◉				◉
Maternal sepsis	Group B Streptococcus		◉									◉		◉
Meningitis	*N. meningitidis*		●		●				◉			◉		◉
	S. pneumoniae		◉		●				◉					◉
	Cryptococcal meningitis	●							◉					
Middle East Respiratory Syndrome coronavirus (MERS-CoV)		●	●	●	●	●	●				◉			
Onchocerciasis		●	●		●		●		◉					◉
Rheumatic fever			●						◉					
Rift Valley Fever (RVF)		●	●	●	●	●	●				◉			
Schistosomiasis		●	●		●		●		◉					◉
Severe Fever with Thrombocytopenia Syndrome (SFTS)		●	●	●	●	●	●				◉			
Sexually transmitted infections (STIs)	Syphilis (incl. congenital syphilis)	◉						●		◉				◉
Soil-transmitted helminthiasis	Hookworm diseases	●	●						◉					◉
	Strongyloidiasis	●	●		●				◉					◉
	Trichuriasis	●							◉					◉
	Ascariasis	●							◉					◉
Taeniasis/cysticercosis		●					●		◉					◉
Trachoma			●		●				◉					◉
Tuberculosis		●	●	●	●				◉			◉		◉
Viral hepatitis (B and C)	Hepatitis C (genotypes)	◉	●		●				◉					◉
Zika		●	●	●	●	●	●				◉	◉		

● Gap identified

Definition: High-priority product gap identified for the disease, condition or pathogen on one or more of the R&D Priority Lists.

◉ Specific gap

Definition: Specific product gap identified, e.g., for a new route of administration to be developed, or serotypes to be targeted.

Priority pathogens

12 pathogens have been brought into the disease scope for the 2018 Index R&D analysis. These have been identified by the WHO pathogen priority list. Pathogens on this list are deemed by WHO as priority R&D targets for new and effective antibiotics active against the pathogens themselves and the diseases they cause. This WHO pathogen priority list does not define specific products needed.

Pathogens	Policy Cures Research G-FINDER neglected diseases	Policy Cures Research G-FINDER reproductive health areas	WHO R&D Blueprint	WHO Initiative for Vaccine Research gaps	WHO Pathogen Priority List	Also analysed in other TAs
Acinetobacter baumannii (carbapenem-resistant)					●	
Campylobacter (fluoroquinolone-resistant)					●	
Enterobacteriaceae (carbapenem-resistant, 3rd generation cephalosporin-resistant)	●				●	●
Enterococcus faecium (vancomycin-resistant)					●	
Haemophilus influenza (ampicillin-resistant)					●	●
Helicobacter pylori (clarithromycin-resistant)					●	
Neisseria gonorrhoeae (3rd generation cephalosporin-resistant, fluoroquinolone-resistant)					●	●
Pseudomonas aeruginosa (carbapenem-resistant)					●	
Salmonella (spp., fluoroquinolone-resistant)	●				●	●
Shigella (spp., fluoroquinolone-resistant)	●				●	●
Staphylococcus aureus (methicillin-resistant, vancomycin intermediate and resistant)					●	
Streptococcus pneumonia (penicillin-non-susceptible)	●				●	●

● Specific gap

Definition: Specific product gap identified, e.g., for a new route of administration to be developed, or serotypes to be targeted.

General notes

Additional to the above diseases and specific targets, the priority lists also include non-specific diseases (multiple or other) which are not further defined.

In some cases of duplicates (an R&D gap has been identified on more than one list) one list may define specific restriction for this gap. The ATMI will consider projects targeting either the general gap or restricted gap equally.
